# Application analysis of transfected cell method for detecting AChR antibodies in MG patients

**DOI:** 10.1038/s41598-026-38414-w

**Published:** 2026-02-10

**Authors:** Wei Liu, Zhenmin Xia, Junyong Hu, Gaijuan Liu, Lijing Zhou, Longlong Xing, Yaxin Qiang, Guanting Lv

**Affiliations:** 1 Hebei Gene-Health Biochip Tech. Co., Ltd, Shijiazhuang, 050035 Hebei Province China; 2 Hebei Jianhai Medical Laboratory, Shijiazhuang, 050035 Hebei Province China; 3 Hebei Yiling Hospital, Shijiazhuang, 050000 Hebei Province China

**Keywords:** Acetylcholine receptor (AChR), Autoantibody, Myasthenia gravis (MG), Ptosis, Transfected cell, Indirect immunofluorescence (IIF), Cell biology, Immunology, Neuroscience, Medical research, Neurology

## Abstract

This study aimed to establish a technical process for detecting nicotinic acetylcholine receptor (nAChR) antibodies using the transfected cell method and evaluate its application in the serological diagnosis of myasthenia gravis (MG), thereby enhancing diagnostic efficiency. Cell transfection technology was used to introduce various nAChR subunit combinations into HEK293 cells for antibody detection. Indirect immunofluorescence (IIF) was utilized to test nAChR antibodies in serum samples from 85 MG patients, and the results were compared for consistency with those of enzyme-linked immunosorbent assay (ELISA).The combination of fetal and adult AChR subunits in transfected cells exhibited the highest sensitivity for detecting serum antibodies in patients with MG. The prepared cell slides demonstrated excellent consistency with the ELISA kit results for 85 MG patients, yielding a Kappa value of 0.769, indicating excellent agreement between the two methods. Co-transfection of multiple AChR subunits successfully generated a cell expressing clustered nAChRs, establishing a highly sensitive detection technique for nAChR antibodies. This technique is invaluable for serological detection of patients with MG, facilitating early disease detection, condition assessment, and therapeutic guidance.

**Supplementary Information:**

The online version contains supplementary material available at 10.1038/s41598-026-38414-w.

## Introduction

Myasthenia gravis (MG) is an autoimmune disorder characterized by autoantibodies that disrupt neuromuscular transmission, leading to clinical manifestations of muscle weakness, fatigue, and fluctuating symptoms throughout the day^1^. The production of acetylcholine receptor antibodies (AChR-Abs) is intimately linked to the pathogenesis of MG because these antibodies target a crucial component of the neuromuscular junction^2^. Approximately 85% of patients with generalized MG and 65–70% of those with ocular MG have detectable AChR antibodies in their serum^3^. Accurate detection of these antibodies in serum is essential for the diagnosis, treatment assessment, and monitoring of MG. However, current detection methods have limitations, with up to half of ocular MG patients and 10 to 15% of generalized MG patients exhibiting negative results in serum nAChR antibody tests^4^. This limitation may be attributed to the low affinity of nAChR antibodies for the receptor, which can potentially lead to undetected cases in standard assays.Given the significance of AChR-Abs in MG and the limitations of current detection methods, there is a compelling need for improved diagnostic approaches. Such advancements could enhance the diagnostic accuracy, enable more effective treatment strategies, and improve the overall management of this debilitating condition.

Clinical investigations have revealed that patients with MG exhibit clinical manifestations and thymic abnormalities comparable to those testing positive for nAChR antibodies. This observation raises the possibility that low binding affinity of these antibodies to the receptor may lead to undiagnosed cases^5^. Original work by the Oxford group demonstrated that live CBA achieved ≥ 90% sensitivity in generalised MG^6,7^.

Considering the advantages of the CBA technique, this study aimed to develop and refine an in-house CBA protocol. We systematically assessed various gene transfection combinations to enhance the sensitivity and specificity of the antibody detection. Additionally, we conducted a thorough comparative evaluation to ascertain the consistency between the CBA and enzyme-linked immunosorbent assay (ELISA) methods to identify nAChR antibodies at the neuromuscular junction of patients with MG.

## Materials and methods

### Experimental materials and reagents

#### Cell lines and plasmids

The human embryonic kidney (HEK293) cell line (Wuhan Shangen Biotechnology Co., Ltd.) was used for heterologous expression of muscle nAChR subunits.

All subunits were cloned into pcDNA3.1(+) vectors containing a CMV promoter and an ampicillin-resistance marker. To monitor transfection, only the Rapsyn was inserted in-frame into the C-terminal eGFP-encoding pcDNA3.1(+)-C-eGFP vector; α1,β1, δ, γ,ε, were expressed from untagged pcDNA3.1(+) plasmids.

#### Reagents

Plasmid DNA was extracted using a kit from Tiangen Company(DP118-02), which ensures endotoxin-free preparation.Lipfectamine 2000 reagent from Thermo Fisher(11668019) .Agarose used for gel electrophoresis was sourced from Biowest Company (A620014-0100). Cell culture media and fetal bovine serum were procured from the Hyclone Company(C11995500BT). Cy3-labeled anti-human IgG from Jackson ImmunoResearch Company(109-165-003) was used as the secondary antibody for immunofluorescence assays.For comparative immunoassays, an enzyme-linked immunosorbent assay (ELISA) kit from the RSR Company was employed(RBA/25).

#### Serum samples

- *MG patient group:* A cohort of 85 serum samples was collected from patients diagnosed with MG at Hebei Yiling Hospital between January 2023 and June 2024.The diagnostic criteria comply with the requirements of the “Chinese Guidelines for the Diagnosis and Treatment of Myasthenia Gravis,” which means that based on the typical clinical features of MG, such as fluctuating muscle weakness, a diagnosis can be made if one of the following tests is abnormal: pharmacological tests, electrophysiological examinations, or serum anti-AChR antibody tests. The demographic distribution included 42 males and 43 females, with an average age of 54.79 years (± 18.62 standard deviation).
- *Pediatric and adult subsets:* Subsets of 12 pediatric cases under the age of 12 and 12 adult cases aged between 30 and 70 years were identified, all with antibody positivity confirmed by ELISA.

The study was reviewed and approved by the Ethics Committee of Hebei Yiling Hospital (Approval No.: 2023LCKY-041-02). All procedures were conducted in accordance with ethical standards, and informed consent was obtained from all participants to ensure their privacy and autonomy.All experimental protocols were conducted in accordance with the relevant guidelines and regulations approved by this committee.

#### Equipment

State-of-the-art equipment, including CO_2_ incubators from HealForce, biosafety cabinets from HFsafe1200LC, ultra-clean workbenches from SW-CJ-2D, constant-temperature shaking incubators from ZHCHENG, water-jacketed incubators from BioPard, and fluorescence microscopes from OLYMPUS BX51, were utilized to maintain optimal experimental conditions.

## Methods

### Gene synthesis and cloning

mRNA sequences encoding human AChR subunits α1 (P3A subtype), β1, γ, δ1, and ε were retrieved from the NCBI database and synthesized by the GenScript Corporation. The synthesized genes were cloned into the pcDNA3.1, vector for expression studies. The Rapsyn gene, essential for AChR clustering, was also optimized to prevent mRNA secondary structures and eliminate immunogenic CpG motifs and labile RNA elements.

### Plasmid preparation and verification

Recombinant plasmids were extracted from the transformed E. coli TOP10 cells using an endotoxin-free plasmid extraction kit. The integrity and orientation of the inserted genes were verified through double enzymatic digestion with BamHI(KpnI)and EcoRI, followed by agarose gel electrophoresis and sequencing at the Qingke Biotech Company.

About 200–1000 ng of plasmid was digested at 37 °C for 30–60 min and analyzed on 1% Agarose Gel.

### Cell culture and transfection

HEK293 cells were cultured to 80–90% confluence in a humidified incubator at 37 °C with 5% CO_2_. Transient transfections were performed using Lipofectamine 2000, following the manufacturer’s protocol, to introduce the plasmid mix into cells. Transfection efficiency was monitored by the expression of eGFP from the pcDNA3.1( +)-C-eGFP vector,and the specific transfection combination information is shown in Table 1.

**Table 1:** Transfection combinations.

Group	Plasmid composition
Transfection group 1—α1	AChR-α1, pcDNA3.1(+)-C-eGFP, plasmid ratio 1:1
Transfection group 2—adult	α1, β1,δ1 , ε, Rapsyn(eGFP) , plasmid ratio 2:1:1:1:2
Transfection group 3—fetal	α1, β1, γ, δ1, Rapsyn(eGFP), plasmid ratio 2:1:1:1:2
Transfection group 4—full combination	α1, β1, γ, δ1, ε, Rapsyn(eGFP), plasmid ratio 2:1:1:1:1:2

### Cell fixation, permeabilization, and blocking

Transfection was performed in 6-well plates. After 5 h, the transfection mix was replaced with fresh complete medium and the cells were cultured for a further 24 h. They were then gently trypsinized and re-seeded (24-well plates, poly-L-lysine-coated coverslips) and allowed to adhere for 24–36 h; transfection efficiency under these conditions is 30–50%.For storage and downstream use, cells were fixed with 4% paraformaldehyde, washed (3 × PBS), and blocked with 3% BSA. The resulting coverslips can be packaged as ready-to-use, room-temperature-stable “cell-on-slide” kits.

### Immunofluorescence assay

Positive and negative serum samples (pre-screened by ELISA) were diluted 1:10 in PBS-1% BSA and incubated on transfected cell coverslips for 1 h at 37 °C. After three washes with PBS, bound antibodies were detected with Cy3-conjugated goat anti-human IgG (diluted 1:500 in PBS-1% BSA) for 1 h at 37 °C. The coverslips were then washed three times with PBST (0.05% Tween-20 in PBS), counter-stained with DAPI for 5 min at RT.

#### Fluorescence scoring criteria

The fluorescence signal intensity was evaluated by two independent investigators in a blinded manner. Based on the brightness and distribution of the red fluorescence (Cy3), the staining was semi-quantitatively scored into four levels:

−, no visible fluorescence;
-  +, weak and sparse fluorescence;
- ++, moderate and patchy fluorescence;
- +++, strong and diffuse fluorescence.

Representative images for each score were captured using consistent microscopy settings (200 × magnification, 500 ms exposure). Discrepancies were resolved by a third senior investigator.

### Age-specific detection analysis

To assess the impact of age on antibody detection, serum samples from 12 juvenile and 12 adult patients with MG were tested using optimized cell slides. The results were analyzed to determine whether age influenced the antibody detection efficacy.

### Clinical sample validation

A total of 85 serum samples from patients with MG were subjected to both the in-house transfection cell method and commercial ELISA kits. Discrepancies in the results were resolved using radioimmunoassay, which served as the gold standard for antibody detection.

### Statistical analysis

Data obtained from both methodologies were statistically analyzed using SPSS 29 software. Concordance between the two methods was assessed using the kappa statistic, with a kappa value ≥ 0.75, indicating excellent agreement.

## Results

### Successful construction of AChR subunit expression vectors

Plasmid vectors containing the human AChR subunit genes were successfully constructed and confirmed through double enzymatic digestion with restriction enzymes BamHI/EcoRI and MluI/EcoRI. The resulting fragments were resolved on a 1% agarose gel, revealing bands corresponding to the anticipated sizes of AChR subunit insertions.The electrophoresis results are shown in Table 2.

**Table 2:**
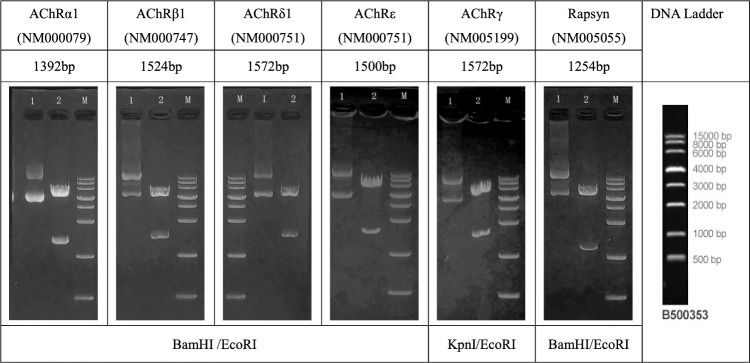
Enzyme digestion electrophoresis diagram.

Lane M: 15000 bp DNA Ladder Lane 1: plasmid. Lane 2: plasmid digested by BamHI(KpnI)and EcoRI. (Full-length blot images are provided in Supplementary Figure).

### Combination-type AChR can accurately distinguish samples from MG patients

**Fig. 1:**
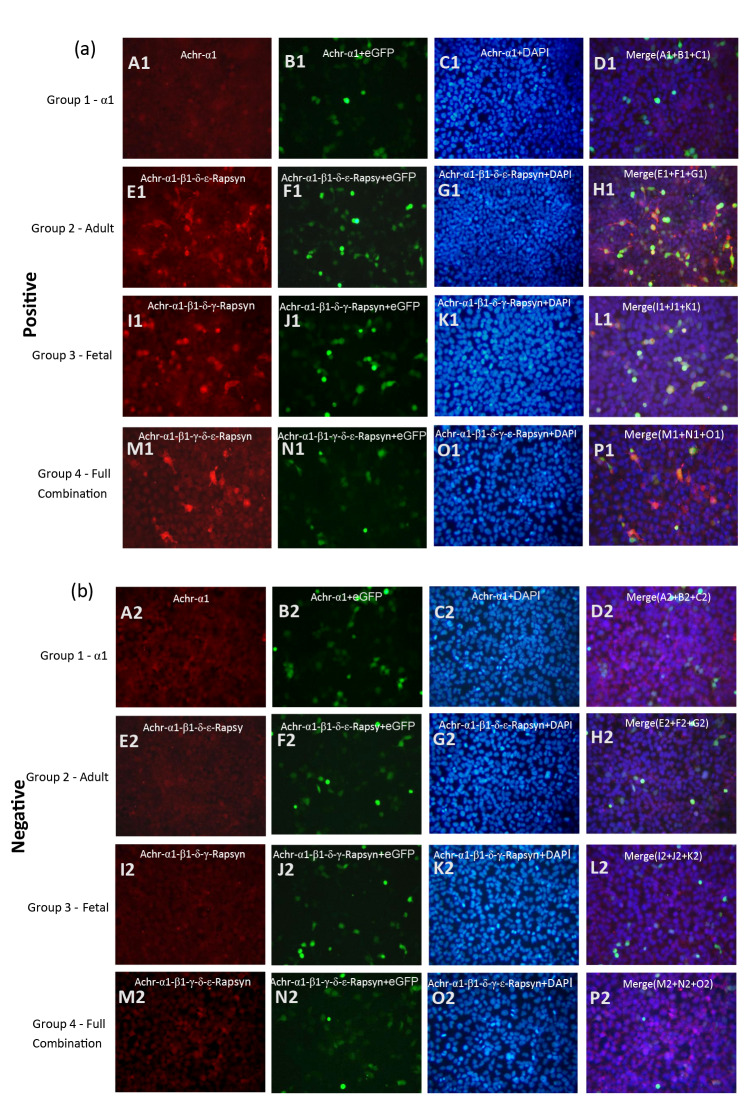
Sample validation of the different transfection combinations. (A1–D1): α1-only transfection, no red fluorescence → α1 alone does not bind serum AChR-abs; E1–H1: adult (α1β1δε) transfection, red fluorescence → abs recognize adult AChR; I1–L1: fetal (α1β1γδ) transfection, red fluorescence → abs recognize fetal AChR; M1–P1: full (α1β1γδε) transfection, red fluorescence → abs recognize adult–fetal hybrid AChR. (A2–P2: No red fluorescence was observed with any transfection format when serum lacking AChR antibodies (ELISA-negative) was tested, confirming assay specificity.)

Serum samples from patients with MG that tested strongly positive using the AChR-ab kit (ELISA method) were selected as positive samples for cell slide detection.Healthy physical examination samples that tested negative using the AChR-ab (ELISA method) kit were selected as negative for cell slide detection. The detection results are shown in Fig. 1. Green fluorescent cells represent cells that have been successfully transfected and express the corresponding proteins, blue represents cell nuclei stained with DAPI, and red represents Cy3-labeled anti-human IgG secondary antibodies.

Figures A1–D1 show that cell slides transfected with only the α1 subunit and reacted with positive samples did not exhibit red fluorescent cells, indicating that the α1 subunit alone can not bind to AChR antibodies in the samples.

Figures E1–H1 show that cell slides transfected with the adult-type (α1, β1,δ1, ε) subunit combination and reacted with positive samples exhibited red fluorescent cells, corresponding to cells expressing eGFP, indicating that AChR antibodies in the samples can bind to the adult-type subunit combination.

Figures I1–L1 show that cell slides transfected with the fetal-type (α1, β1, γ, δ1) subunit combination and reacted with positive samples also exhibited red fluorescent cells, corresponding to the cells expressing eGFP, indicating that AChR antibodies in the samples can bind to the fetal-type subunit combination.

Figures M1–P1 show that cell slides transfected with the full adult-fetal combination and reacted with positive samples similarly exhibited red fluorescent cells, corresponding to the cells expressing eGFP, indicating that AChR antibodies in the samples can bind to the full adult-fetal combination.

**Fig. 2:**
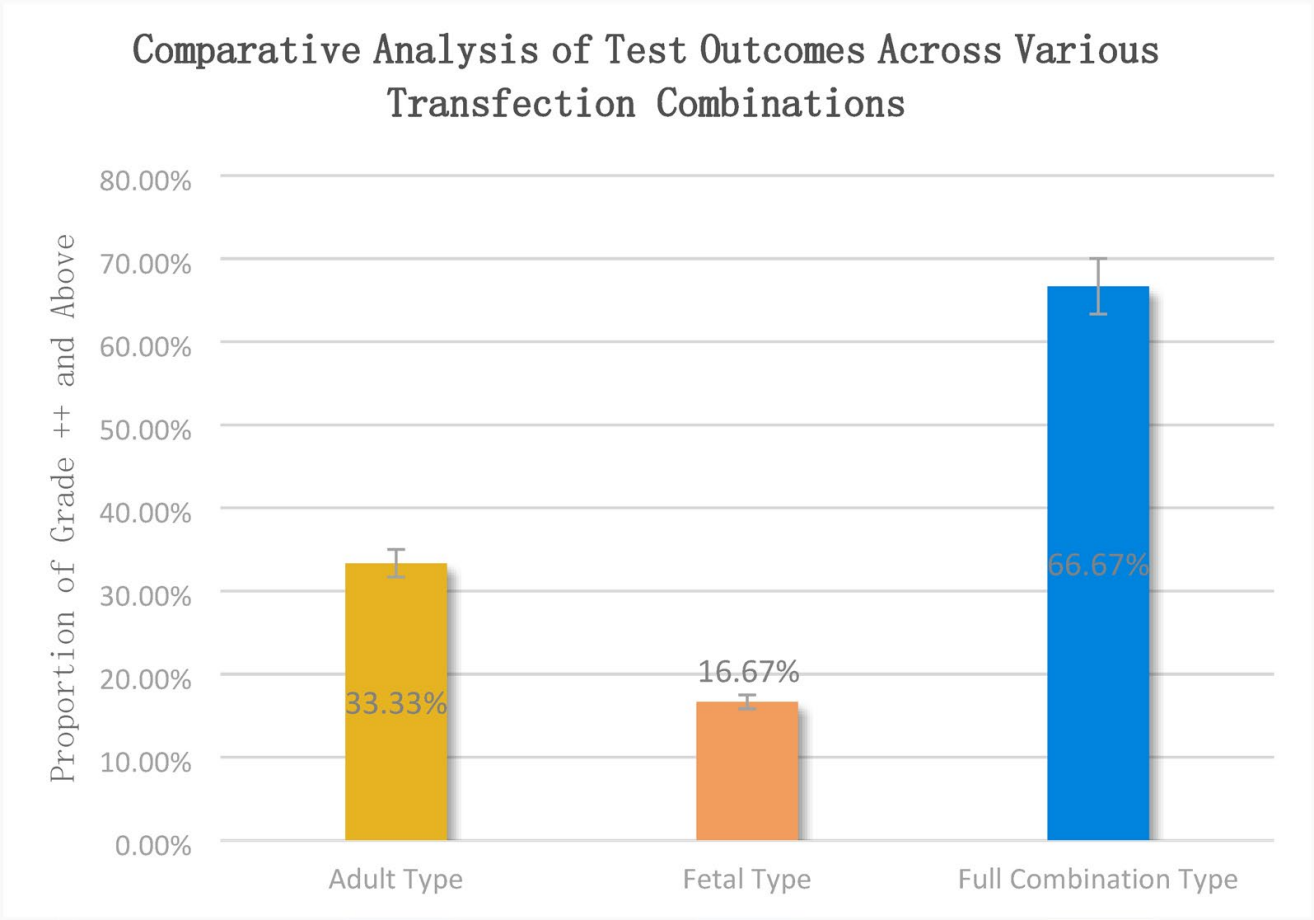
Comparative of detection results from different transfection combinations.

In the reaction with negative samples, no significant red fluorescent cells were observed for any of the combinations (A2–P2) . To further verify the effectiveness of cell slides transfected with the AChR-α1 subunit in binding to AChR antibodies in serum, we collected 48 samples that tested positive for antibodies by ELISA and conducted cell-based antibody detection. The results showed that No fluorescence was observed in any of the samples, indicating that cells expressing the AChR-α1 subunit can not be used for the detection of serum AChR antibodies. (Fig. 2)

### Full combination-type AChR significantly enhances the detection rate of positives

For the 12 juvenile (age < 12 years) and 12 adult samples (age: 30–70 years) selected in this study, the detection results of different transfection combinations were analyzed. The results are presented in the Table 3.

**Table 3:** Differences in detection results with different transfection combinations.

Group	Sample	Age/gender	Elisa result	Group 2—adult Type (α1, β1, δ, ε)	Group 3—fetal type (α1, β1, γ, δ)	Group 4—full combination type (α1, β1, γ, ε, δ)
Juvenile	M1	8/female	0.82	+	+	+
	M2	7/female	1.43	+	( +)	+
	M3	8/female	1.79	+	–	+
	M4	12/male	2.62	+	+	++
	M5	5/female	3.61	+	+	++
	M6	4/male	4.26	+	++	++
	M7	2/female	4.63	+	(+)	+
	M8	12/male	6.44	+	+	++
	M9	3/female	6.76	++	+	++
	M10	9/male	7.21	(+)	++	++
	M11	10/female	11.05	++	+	++
	M12	5/female	25.15	++	+	+++
Adult	A1	42/male	1.05	+	+	+
	A2	27/female	2.21	+	+	+
	A3	42/male	7.45	+	+	+
	A4	73/female	11.45	+	(+)	+
	A5	40/female	14.61	+	+	++
	A6	24/female	17.61	+	+	++
	A7	25/male	18.64	++	+	++
	A8	48/female	23.65	+++	+	+++
	A9	42/male	24.44	++	+++	+++
	A10	69/female	26.52	+	++	+++
	A11	72/male	28.14	+++	+	+++
	A12	43/male	28.46	+++	+	+++

				Group 2—adult type (α1, β1, γ, ε)	Group 3—fetal type (α1, β1, γ, δ1)	Group 4—full combination type (α1, β1, γ, ε, δ1)
Statistical percentages				+++ is observed in 12.5% (3/24) ; ++ is observed in 20.83% (5/24) ; + is observed in62.5% (15/24) ; (+) is observed in 4.17% (1/24)	+++ 4.17% (1/24), ++ is observed in 12.5% (3/24), + is observed in 66.67% (16/24), (+) is observed in 12.5% (3/24), - is observed in 4.16% (1/24)	+++ is observed in 25% (6/24), ++ is observed in 41.67% (10/24), + is observed in 33.33% (8/24)

Comparing the detection results, we observed that cell slides transfected with Transfection Group 4 ( full combination type, including α1, β1, γ, ε, and δ1 subunits) demonstrated the most pronounced fluorescence intensity. Particularly, in samples with lower antibody titers, where the cell slides from Transfection Group 3 (fetal type α1, β1, γ, δ) failed to detect cellular fluorescence, the cell slides from Transfection Group 4 exhibited a more noticeable fluorescent signal. From a comprehensive assessment of the 24 samples, the cell slides from Transfection Group 4 showed a significant advantage in detecting fluorescence intensity, with a notable or stronger fluorescence signal (marked as “++” or above) observed in 66.67% of the samples. In comparison, the proportions in Transfection Groups 3 and 2 were 16.67% and 33.33%, respectively. These data indicate that Transfection Group 4 had a significant detection advantage over groups 2 and 3, especially in detecting samples with low antibody titers. Additionally, the fetal type combination and the adult-type combination did not demonstrate any correlation in detection specific to age groups.

### Cell-based assay (CBA) outperforms traditional ELISA in clinical sample testing

A total of 85 patients with MG were included in this study. Enzyme-linked immunosorbent assay (ELISA) using the AChR-Ab ELISA kit of the RSR company detected 58 patients (68.24%) with positive anti-acetylcholine receptor (AChR) antibodies, while 27 patients (31.76%) were seronegative. Cell-based assay (CBA) results indicated that 64 patients (75.29%) were positive for anti-AChR antibodies and 21 patients (24.71%) were seronegative. All 57 ELISA-positive samples exhibited clear immunofluorescence in the CBA; one ELISA-positive sample was not detected by the CBA, and seven samples were found to have low-affinity AChR antibodies.

After conducting a thorough analysis of the data obtained from both testing methods using the Statistical Package for the Social Sciences (SPSS) data analysis software, this study employed McNemar’s test for paired differences. The analysis revealed no statistically significant difference in the overall negative and positive rates between the two methods (*P* = 0.07, where a *P*-value greater than 0.05 indicates a non-significant difference). Subsequently, a consistency test yielded a kappa value of 0.769 with a *p*-value less than 0.001, indicating a high level of concordance between the two testing methods;the results are presented in Table 4 below.

**Table 4:** Data statistics comparison.

Method 1 (CBA)	Method 2 (ELISA)		Total	McNemar’s Test
	Positive	Negative		
Positive	57	7	64	*P*=0.07（not significant if >0.05）
Negative	1	20	21	
Total	58	27	85	
Kappa coefficient (95% CI)	0.769（*P*<0.001）			

Radioimmunoassay (RIA) was used for comparative analysis of the discordant sample results. Among the eight discordant results, the CBA findings were consistent with the RIA results in six cases, suggesting that the CBA may demonstrate superior detection performance compared with the ELISA method in certain instances.

Based on this analysis, we conclude that CBA, as an emerging detection technique, is consistent with the diagnostic performance of commercial kits and may provide more accurate results in specific situations. This finding provides robust statistical support for the potential application of CBA in clinical diagnoses.

## Discussion

The nAChR, situated at the neuromuscular junction, is a ligand-gated ion channel critical for rapid signal transduction between spinal motor neurons and muscle cells and is a principal antigenic target in MG^7^. In adult mammalian muscles, nAChR is composed of a pentameric structure featuring two α1, β1, δ, and ε subunits, whereas in the fetal form, the ε subunit is substituted by the γ subunit^8^.Adult muscle nAChR forms a cylindrical ion channel complex in the order α1-ε-α1-δ-β1. Extensive research has confirmed the significant role of anti-α1 antibodies in the pathogenesis of MG^9^. The main immunogenic region (MIR) of nAChR antibodies is located on the α1 subunit, which is the predominant site for stimulation of nAChR antibody production in MG patients^10^. Despite the α1 subunit harboring the primary immunogenic region, autoantibodies against other subunits are also present in patients^11^. Antibodies against the nAChR-α1 subunit exhibit greater pathogenicity than those against the β1 subunit^12^, and γ subunit-specific autoantibodies can lead to arthrogryposis in neonates and recognize nAChRs in extraocular muscles in adults^13^. To enhance diagnostic precision, existing detection technologies include radioimmunoassay (RIA), which primarily utilizes AChR antigen purified from human muscle tissue and human rhabdomyosarcoma TE671 cells, and enzyme-linked immunosorbent assay (ELISA), which employs fetal and adult AChRs as antigens. The cell-based assay (CBA) for nAChR antibody detection co-transfects α1, β1, δ, ε, or γ subunits with the Rapsyn gene to emulate the clustered expression of the receptor on the postsynaptic membrane in vivo^14^, thereby achieving efficient detection of antibodies present in the body.

In this study, our initial transfection of the AChR-α1 subunit into HEK293 cells for serum antibody detection anticipated that some samples would exhibit a negative reaction due to the expression of antibodies against subunits other than α1. However, experimental results revealed that none of the 48 antibody-positive samples specifically bound to cells expressing the α1 subunit. This finding prompted a reevaluation of the relationship between nAChR conformation and antibody-binding affinity. When HEK293 cells were transfected with adult- and fetal-type subunit combinations, serum antibodies effectively bind, suggesting that the clustered nAChR may possess a unique conformation. Researchers, such as Kaori Noridomi, have noted that subtle structural differences between the monomeric and pentameric forms of mammalian nAChRα1 could affect binding affinity^7^. The monomeric α1 subunit may exhibit higher kinetic activity than pentameric nAChR, whereas the fully assembled pentameric nAChR may be structurally more compact with reduced conformational flexibility, potentially diminishing binding affinity through an entropic effect. This may explain why no fluorescence signal was detected in the 48 positive samples in our experiment. Additionally, previous studies, including those by Newland et al. in 1995, have identified two splice variants of the AChR α1 subunit in human muscle, one consisting of 437 amino acids and the other containing an additional 25 amino acids in the N-terminal extracellular domain^15^. The human muscle cell line TE 671 was found to have a ratio of approximately 1:1 for the two α1 subunits. This study used a shorter splice variant, which may account for the lack of antibody binding. Future experiments will test the combination of both splice variants to explore potential differences in antibody binding, furthering our understanding of nAChR structure and function, and its role in MG pathogenesis.

In addition,although the α1 subunit harbours the main immunodominant region (MIR) for pathogenic IgG, its ability to bind serum antibodies in situ depends on whether it reaches the plasma membrane. Solution-phase studies have shown that recombinant α1 extracellular domain (α1-ECD, aa 1-210) quantitatively adsorbs AChR antibodies from patient sera, reducing RIA counts by > 90%^6^. These data establish that the MIR epitopes are located exclusively on α1. However, when α1 is expressed alone in intact cells it is retained in the endoplasmic reticulum (ER) because the nAChR pentamer follows a strict “assembly-checkpoint” rule: only α-β and α-δ dimers that incorporate the β, γ or ε partners are exported to the Golgi and subsequently to the surface^16^. Consistent with these trafficking studies, we observed no α-bungarotoxin or patient-IgG staining on live HEK293 cells transfected with α1 alone (present study and Fig. 1. A1). Therefore, the absence of fluorescent signal in our α1-only coverslips reflects failure of the unassembled subunit to reach the cell surface rather than loss of antigenicity of the α1 MIR itself. Once α1 is forced to the membrane—e.g. by fusion to a CD4 trans-membrane domain or by co-expression with the minimum β1 and δ partners—serum antibodies bind with an affinity comparable to that seen for the full pentamer^17^. Taken together, these findings reconcile the apparent contradiction between solution-phase adsorption and cell-based staining, and underscore why a single-subunit CBA is unsuitable for routine antibody detection.

When we compared the detection signals of adult-type vs. fetal-type transfected cell slides, the inclusion of 24 sera (12 pediatric and 12 adult MG patients) showed a step-wise fluorescence intensity: adult + fetal combination > adult alone > fetal alone. Rather than conflicting with the literature, these data mirror the larger survey of 200 MG sera in which the combined use of both AChR isoforms raised the overall antibody-positive rate from ≈ 66% (single isoform) to 77%^13^. What our small cohort adds is a dynamic explanation for why the combined antigen gives higher sensitivity: the “extra” signal comes disproportionately from older patients who are more likely to be “dual-positive”.In the published series, the frequency of antibodies restricted to only the ε (adult) or only the γ (fetal) subunit was similar (22 vs 14 cases), but patients positive for both isoforms were significantly older and showed the highest risk of progression from ocular to generalized MG (OR 5.09, 95% CI 2.23–11.62; independent effect of age OR 1.03 per year). Ageing muscle may re-express fetal-type AChR (γ-subunit) as part of denervation–re-innervation remodelling, while the adult-type (ε-subunit) remains present; the immune system is then exposed to two distinct extracellular domains, doubling the number of potential epitopes. Consequently, the older the patient, the greater the probability that serum will contain a mixed anti-ε/anti-γ repertoire; a single-isoform slide will capture only one subset, whereas a combined adult + fetal transfection captures both, producing the brighter fluorescence we observed.Therefore, the combined-antigen CBA not only increases raw sensitivity but also weights detection toward the very group (higher-age, dual-positive) that carries the highest short-term risk of generalization. Larger prospective studies are needed to confirm whether the quantitative fluorescence intensity on a combined slide can be used as a surrogate marker for both age-related antibody diversity and impending clinical worsening.

Among the current nAChR antibody detection methods, radioimmunoassay (RIA) offers high specificity and sensitivity and serves as the gold standard for anti-AChR antibody detection^18^. However, due to the absence of conformational epitopes of natural AChR clusters, solubilized nAChR in RIPA cannot recognize low-affinity nAChR antibodies in some patients with MG. Leite et al. found low-affinity serum anti-nAChR antibody clusters in 66% of seronegative MG patients^14^, whereas the CBA method can sensitively detect low-affinity nAChR antibodies undetectable by RIA^19^. Enzyme-linked immunosorbent assay (ELISA) has become the most commonly used method owing to the practical limitations of radioactive reagents. In the clinical sample validation phase of this experiment, we compared the application effects of the enzyme-linked immunosorbent assay and cell-based detection methods. Based on the aforementioned comparison results of adult, fetal, and adult and fetal combination types, we used adult and fetal combination cell slides to detect 85 clinical samples to enhance screening efficiency for patients with diverse clinical phenotypes. The detection system constructed in this study demonstrated consistent results with the enzyme-linked immunosorbent assay (concordance coefficient kappa value = 0.769), indicating that the cell-based detection method constructed in this study exhibits highly consistent detection performance with commercial kits. This comparative analysis confirmed the accuracy and reliability of the CBA method as an emerging detection technology for the diagnosis of MG. The research outcomes not only provide a scientific basis for the application of the CBA method in the detection of MG antibodies but also offer new perspectives and potential directions for improvement in future clinical diagnosis and disease monitoring.

Our study has several limitations. First, the absence of non-MG disease controls and samples from healthy donors precluded calculation of true sensitivity, specificity, or cut-off values; we could only compare agreement with ELISA results, which does not establish the diagnostic accuracy of the assay. Prospective, multicentre studies that include large control cohorts are required to define formal performance metrics and optimal thresholds.

Furthermore, studies have reported that live-cell-based nAChR antibody detection (Live-CBA) surpasses the sensitivity and specificity of radioimmunoassay (RIA) and fixed-cell-based assay (Fixed-CBA)^20–22^. In a study of 86 RIA antibody-negative samples, the fixed CBA method detected antibodies in 10 cases (11.6%, 95% CI 5.7–20.3), whereas the live cell CBA method detected antibodies in 16 cases (18.6%, 95% CI 11.0–28.5)^23^. Although Live-CBA exhibits excellent detection efficacy, its widespread adoption is limited by the high professional requirements for detection institutions in cell culture and detection operations. Given the hardware facilities and personnel knowledge reserves of our laboratory, we preliminarily assessed the feasibility of introducing Live-CBA technology. Future research will explore and optimize the live cell antibody detection process to enhance the detection accuracy and efficiency of nAChR antibodies in newly diagnosed MG patients with low antibody titers, providing more reliable scientific evidence for the diagnosis and treatment strategies of MG and offering important clues for in-depth research on disease treatment strategies.

## Supplementary Information

Below is the link to the electronic supplementary material.

Supplementary Material 1

## Data Availability

The experimental raw datasets generated during this study are available in the ScienceDB repository at 10.57760/sciencedb.33610
