# Supplementary material for: Application analysis of transfected cell method for detecting AChR antibodies in MG patients
Source: Sci Rep. 2026 Feb 10;16:8130. doi: 10.1038/s41598-026-38414-w (PMC12960793; doi:10.1038/s41598-026-38414-w)
Supplement: Supplementary file 1 — Supplementary Material 1 [file 41598_2026_38414_MOESM1_ESM.docx]

| 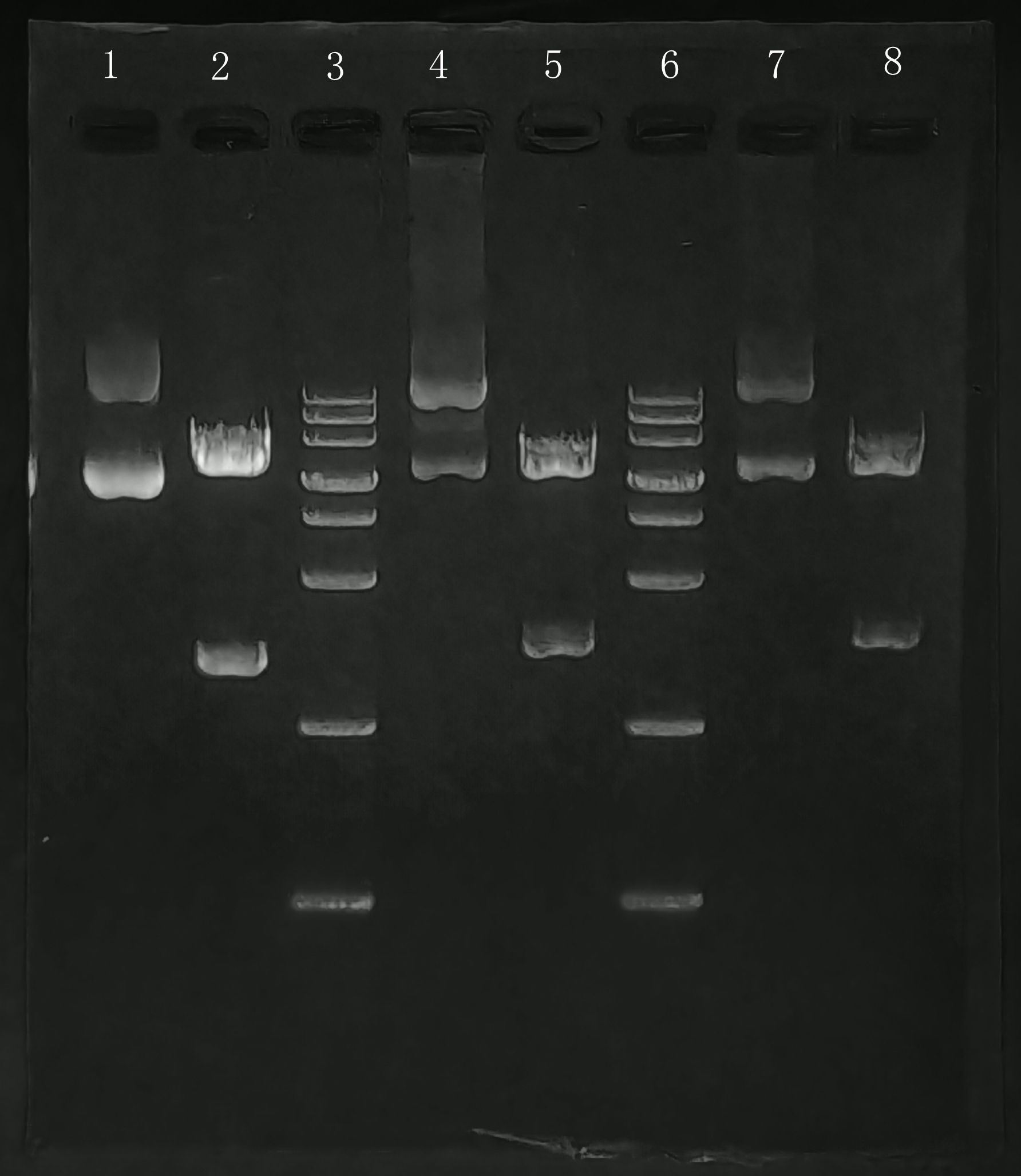  Supplementary Figures1 | Lane1: AchR-a plasmid  Lane2: AchR-a plasmid digested by BamHI and EcoRI  Lane3: 15000bp DNA Ladder  Lane4: AChRβ plasmid  Lane5: AChRβ plasmid digested by BamHI and EcoRI  Lane6: 15000bp DNA Ladder  Lane7: AChRδ plasmid  Lane8: AChRδ plasmid digested by BamHI and EcoRI |
| --- | --- |
| 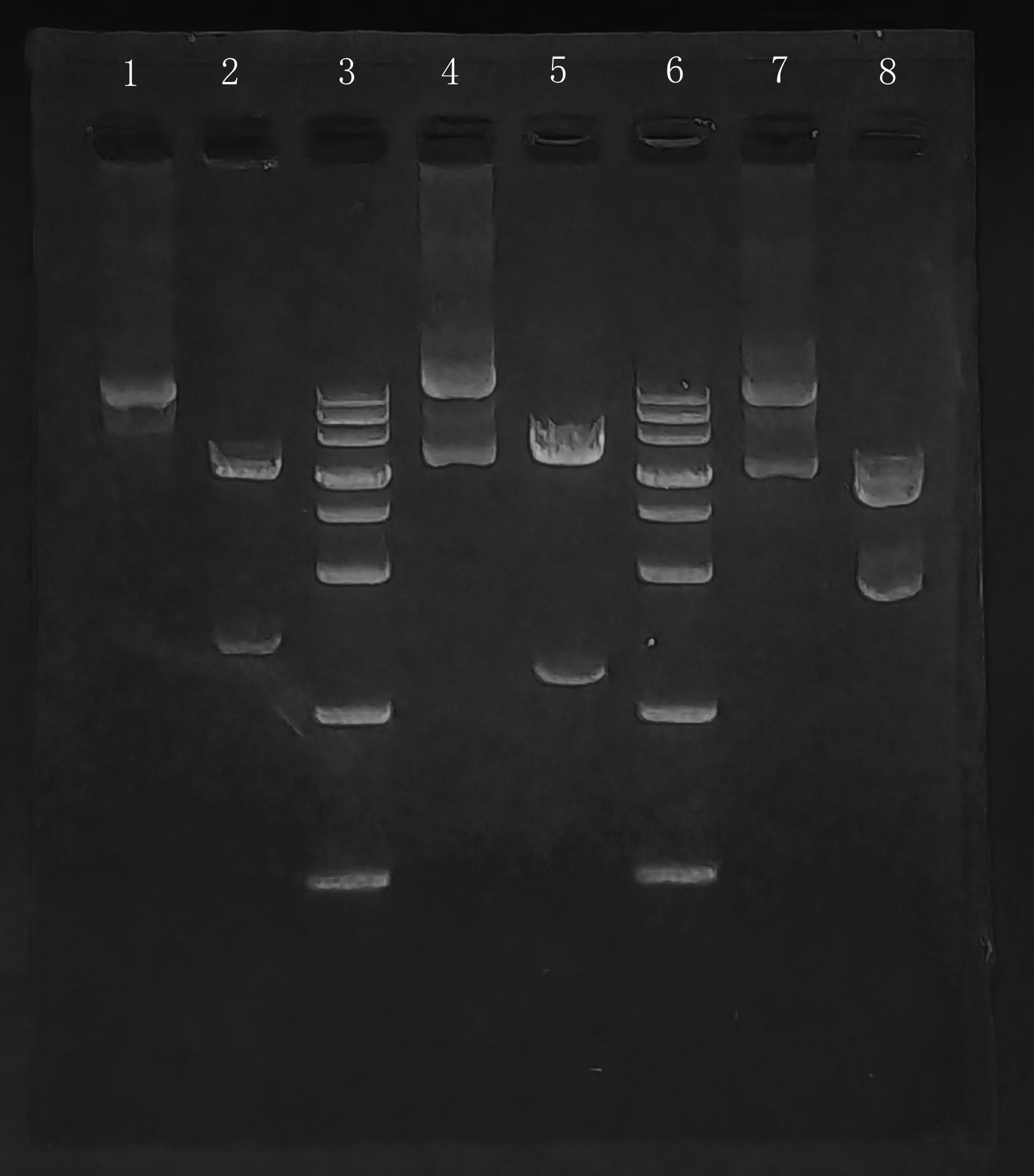  Supplementary Figures2 | Lane1: AchR-ε plasmid  Lane2: AchR-ε plasmid digested by BamHI and EcoRI  Lane3: 15000bp DNA Ladder  Lane4: Rapsyn plasmid  Lane5: Rapsyn plasmid digested by BamHI and EcoRI  Lane6: 15000bp DNA Ladder  Lane7: AChRγ plasmid  Lane8: AChRγ plasmid digested by MluI and EcoRI |
| 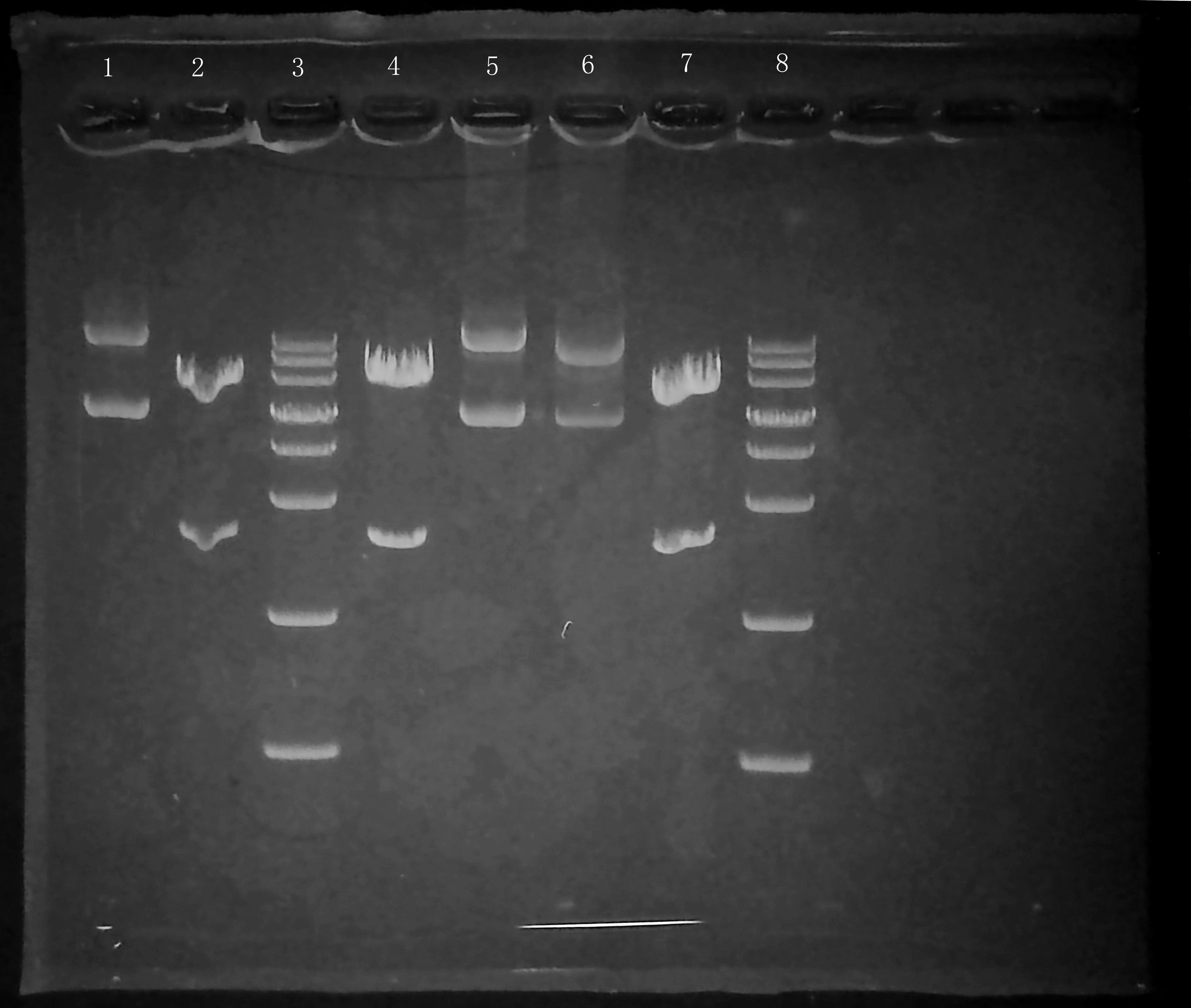  Supplementary Figures3 | Lane1: AChRδ plasmid  Lane2: AchR-δ plasmid digested by BamHI and EcoRI  Lane3：15000bp DNA Ladder  Lane4：AchR-ε plasmid digested by BamHI and EcoRI  Lane5：AchR-ε plasmid  Lane6：AChRγ plasmid  Lane7：AChRγ plasmid digested by KpnI and EcoRI  Lane8：15000bp DNA Ladder |
